# What factors influence a Quality Improvement Collaborative in improving contraceptive services for foreign-born women? A qualitative study in Sweden

**DOI:** 10.1186/s12913-023-10060-2

**Published:** 2023-10-11

**Authors:** Ingrid Siösteen-Holmblad, Elin C. Larsson, Helena Kilander

**Affiliations:** 1 Department of Global Public Health, Karolinska Institutet, Stockholm, Sweden; 2 Department of Women’s and Children’s Health, Karolinska Institutet, and the WHO Collaborating Centre, Karolinska University Hospital, Stockholm, Sweden; 3 Department of Women’s and Children’s Health, Uppsala University, Uppsala, Sweden; 4 Jönköping Academy for Improvement of Health and Welfare, School of Health and Welfare, Jönköping University, Jönköping, Sweden

**Keywords:** Contraception, Consolidated Framework for Implementation Research, Counselling, Family planning, Maternal health care, Postpartum, System performance

## Abstract

**Background:**

Improved contraceptive services could reduce the unmet need for contraception and unintended pregnancies globally. This is especially true among foreign-born women in high-income countries, as the health outcomes related to unmet need of contraception disproportionally affect this group. A widely used quality improvement approach to improve health care services is Quality Improvement Collaborative (QIC). However, evidence on *to what extent*, *how* and *why* it is effective and what factors influence a QIC in different healthcare contexts is limited. The purpose of this study was to analyse what factors have influenced a successful QIC intervention that is aimed to improve contraceptive service in postpartum care, mainly targeting foreign-born women in Sweden.

**Methods:**

A qualitative, deductive design was used, guided by the Consolidated Framework for Implementation Research (CFIR). The study triangulated secondary data from four learning seminars as part of the QIC, with primary interview data with four QIC-facilitators. The QIC involved midwives at three maternal health clinics in Stockholm County, Sweden, 2018–2019.

**Results:**

Factors from all five CFIR domains were identified, however, the majority of factors that influenced the QIC were found inside the QIC-setting, in three domains: intervention characteristics, inner setting and process. Outside factors and those related to individuals were less influential. A favourable learning climate, emphasizing co-creation and mutual learning, facilitated reflections among the participating midwives. The application of the QIC was facilitated by adaptability, trialability, and a motivated and skilled project team. Our study further suggests that the QIC was complex because it required a high level of engagement from the midwives and facilitators. Additionally, it was challenging due to unclear roles and objectives in the initial phases.

**Conclusions:**

The application of the CFIR framework identified crucial factors influencing the success of a QIC in contraceptive services in a high-income setting. These factors highlight the importance of establishing a learning climate characterised by co-creation and mutual learning among the participating midwives as well as the facilitators. Furthermore, to invest in planning and formation of the project group during the QIC initiation; and to ensure adaptability and trialability of the improvement activities.

**Supplementary Information:**

The online version contains supplementary material available at 10.1186/s12913-023-10060-2.

## Introduction

Quality Improvement Collaboratives (QIC) have been used extensively to improve health care globally, including sexual and reproductive health and rights (SRHR) [1–6]. QICs aim to close identified gaps between research and health care for specific clinical challenges and continuously integrate and test changes through a rapid and measurable process [7]. The OIC depart from a pre-identified clinical problem and thereafter participants decide on improvement activities that are implemented during continuous and rapid cycle tests performed by the health care professionals in their specific contexts [7]. Studies on QICs show promising results [1–5], indicating positive outcomes and cost-effectiveness [1, 6]. Evidence suggests that interactive and multidisciplinary teamwork in which participants are empowered to identify, test and evaluate changes, are factors associated with success of the QIC method [1, 6, 8–10]. Furthermore, participants and stakeholders should be committed to the QIC and understand the objectives, and a skilled and well-structured planning team as well as sufficient resources is needed [1, 5–8, 11, 12].

However, several studies have argued that the positive effects of QICs are small and unreliable, and that interpretations on QIC effectiveness should be made with caution [2, 4, 5]. Issues contributing to the weak evidence-base include a lack of understanding on the role of context and which specific components are important to success of QICs [1, 4, 5]. Proposed reasons behind the varying results are lack of adaptation to the clinical context and that QIC’s program theory have been justified based only on successful QICs elsewhere [1, 13]. Since QICs are a common approach to improving quality of care globally, it is therefore essential to understand the factors influencing promising QICs [1–6].

One area in which QICs have been used is contraceptive services [14, 15]. Globally, improved access to contraceptive services has the potential to strengthen sexual and reproductive health and rights, including preventing unintended pregnancies postpartum and facilitating birth spacing [16–18]. Foreign-born women in high- and low-income countries report lower contraceptive use, reflecting high rates of unintended pregnancies and abortions due to factors such as adverse socioeconomic conditions and lack of access to common pathways to healthcare [19–23]. However, postpartum contraceptive services provided to foreign-born women constitute a complex setting and there is a substantial need to understand factors influencing successful quality improvement interventions in this setting. In Sweden, a recent QIC report positive results regarding improved access to effective contraception post-abortion [24]. Effective contraception was defined as either short acting reversible contraception (ie contraceptive pill) or long acting reversible contraception (ie intrauterine devices) [18].

A remaining question is, however, which components of the successful QICs in the Swedish setting that contributed to the improved outcomes [11, 24]. The purpose of this study was to analyse what factors have influenced a successful QIC intervention that is aimed to improve contraceptive service in postpartum care, mainly targeting foreign-born women.

## Methods

A qualitative deductive approach, triangulating secondary and primary data was used to study what factors influenced a QIC, aiming to increase the proportion of women choosing effective contraception postpartum [25]. The study design was guided by the Consolidated Framework for Implementation Research (CFIR) [26].

### Study setting

The QIC analysed in this study took place September 2018 – August 2019 at three Maternal Health Clinics (MHCs) in Stockholm County, Sweden in municipalities with both high and low proportion of foreign-born inhabitants [27]. Performance data were retrieved from the Swedish Pregnancy register (SPR) and has been reported elsewhere [25], showing that the proportion of women choosing a more effective postpartum contraceptive method increased from 30 to 47% among foreign-born women [25]. Midwives expressed that registering performance data was beneficial and that participating in the QIC developed their counselling skills [25].

### Design of the Quality Improvement Collaborative

**Fig. 1:**
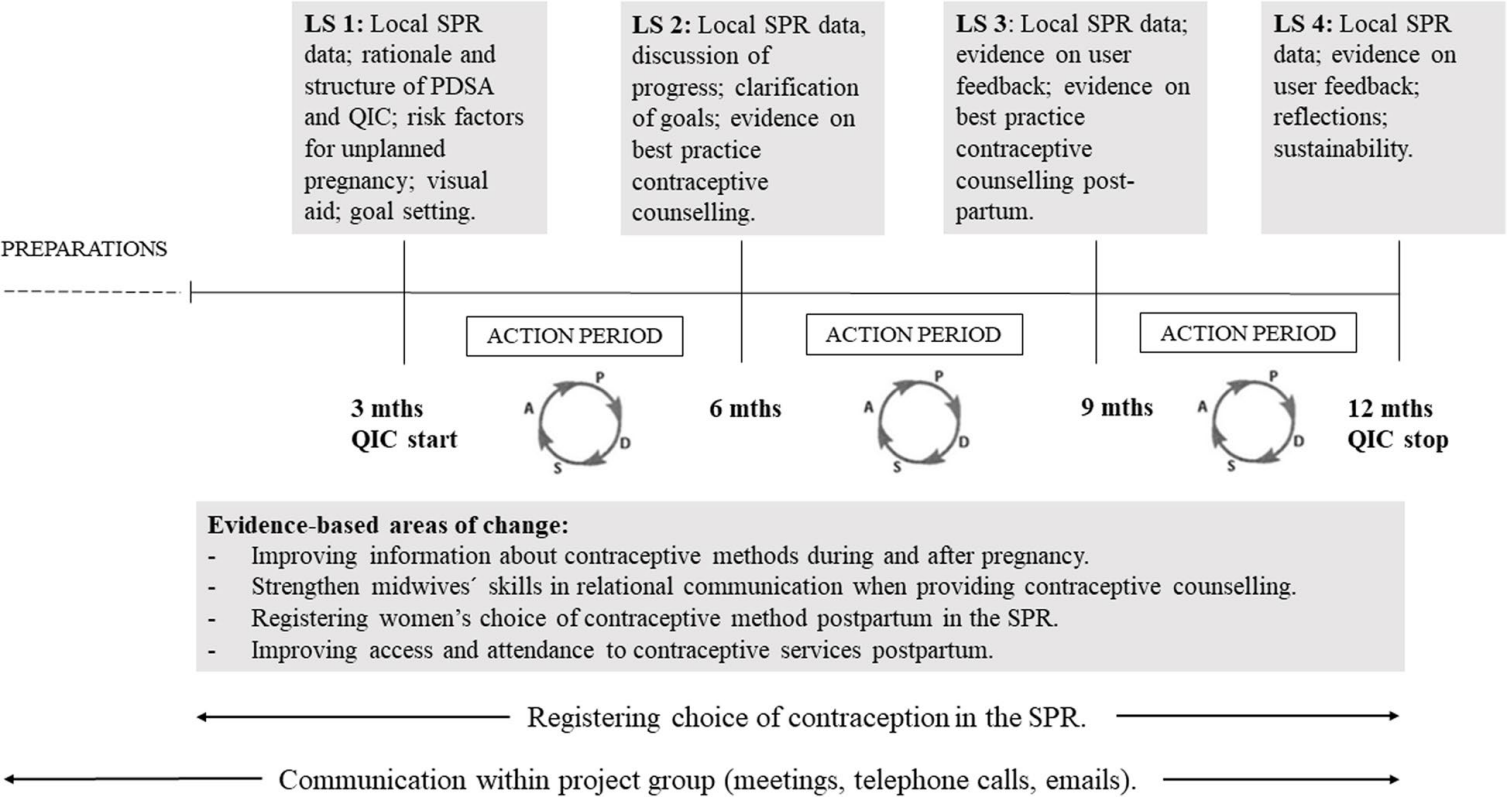
Outline of the Quality Improvement Collaborative in contraceptive services postpartum 2018–2019. LS = Learning Seminar; PDSA = Plan-Do-Study-Act; QIC = Quality Improvement Collaborative; SPR = Swedish Pregnancy Register

The QIC was designed in line with the Breakthrough model [7], using Plan-Do-Study-Act (PDSA) cycles [28] (Fig. 1). The learning seminars (LS) consisted of lectures, sharing experiences of contraceptive counselling, reflections on SPR data such as women’s choice of contraception in relation to maternal health clinic and country of birth, as well as planning of evidence-based improvement activities. At the first learning seminar, participants were introduced to four evidence-based areas presented in a Driver diagram [25]. Participants selected improvement activities within their respective maternal health clinics (Fig. 1). The user perspective on the improvement activities was gathered through interviews with women and men and fed back during learning seminars 3–4. The improvement activities were implemented during action periods between the learning seminars, at which lessons learned or questions regarding implementation was discussed.

### Participants

The QIC project group consisted of five facilitators and the participants were 10–13 midwives employed at the maternal health clinics. Key stakeholders were the heads of the maternal health clinics (*n* = 2) who participated partly in the project (Table 1).

**Table 1:** Overview of data and data collection methods

	**Secondary data**	**Primary data**
Source	Learning seminars (*n* = 4)	Facilitators (*n* = 4)
Type of data	Transcribed recordings from LS, 220 pages	Transcribed semi-structured interviews, 51 pages
Participants	Midwives (*n* = 10–13 in each LS) Heads of MHCs	4 out of 5 facilitators from the QIC project group: - Project leader - Principal Investigator - Improvement Advisor for maternal health care (coordinator) - Improvement Advisor supporting the SPR - Research Assistant supporting planning, data collection and analysis
Point in time	Dec 2018 – Oct 2019	Feb – March 2021

*LS* Learning Seminar, *MHC* Maternal Health Clinic, *QIC* Quality Improvement Collaborative, *SPR* Swedish Pregnancy Register

### Conceptual framework

The CFIR was used for the analysis of this study [26]. The CFIR syntheses constructs from published implementation theories and compiles them into a meta-theoretical framework consisting of five domains: intervention characteristics, inner setting, outer setting, individual characteristics, and process, together covering 39 defined constructs (see Additional file 1) [26].

### Data collection and analysis

Secondary qualitative data was collected during the QIC (Table 1) by recording all learning seminars (*n* = 4) conducted during the QIC. The recordings were transcribed verbatim by author ISH.

Primary qualitative interview data was collected after the QIC had concluded (Table 1). For the primary data collection, a semi-structured interview guide (see Additional file 2) was developed based on the analysis of secondary data and guided by the CFIR Interview Guide Tool [29]. These interviews were conducted after the analysis of the secondary data in order to complement and follow up on issues not covered. Online interviews were conducted by author ISH with four of the five facilitators who worked directly with the QIC. The interviews lasted 45–60 min and were recorded and transcribed verbatim by author ISH. Lastly, a member check was performed with all respondents.

Both secondary and primary data was analysed using thematic analysis [30], with a deductive approach informed by the CFIR [26]. The 39 pre-defined *constructs* of the framework were used as codes and the five domains as themes. Analysis was conducted by author ISH, using the pre-defined CFIR-constructs in a reflexive manner perceptive to nuances, divergencies and how “sub-constructs” within different constructs related to each other [31]. The final results were discussed among all three authors until consensus was reached.

### Ethical considerations

The organisational case study had ethical approval from the Regional Ethics Committee in Stockholm, ref: 2017/1312–31/5; 2108/1241–32.

## Results

The majority of CFIR constructs that influenced the successful QIC were found within the domains Inner setting, involving the maternal health clinics or the project group, Process i.e. factors related to the different stages of the QIC and Intervention characteristics, i.e. key attributes of the QIC (Table 2). Outer setting involved factors that have an origin outside of the maternal health clinics or the project group, and Individual characteristics focused on the specific participants. An overview of results is presented in Table 2.

**Table 2:** The identified constructs within their respective CFIR-domains and a summary of results

CFIR Domain	Inner setting	Process	Intervention characteristics	Outer setting	Individual characteristics
**CFIR Constructs**	***Learning climate*** – a favourable learning climate was created in which mutual knowledge sharing was emphasized and holistic solutions created	***Project group***^***a***^ – engaged and skilled facilitators in the project group contributed to success of the QIC	***Co-creation***^***b***^ – the QIC was developed by both midwives and stakeholders, as well as by the project group	***Patient needs and resources*** among the foreign-born women brought up different perceptions among midwives on how to approach their needs	***Knowledge and beliefs*** – knowledge of QIC’s was limited among midwives, but professional skills facilitated application of improvement activities
	***Communication*** – worked well at LS and within the project group but was limited between the facilitators and midwives during action periods	***Planning*** – overall good planning which facilitated the QIC, but insufficient anchoring among stakeholders was a barrier	***Adaptability*** – was integral to the PDSA structure and facilitated application of the QIC and inclusion of participant’s input		
	***Information and knowledge*** – knowledge exchange in three directions was a facilitating factor at LS	***Engaging*** – efforts were put into creating an engaging atmosphere at LS. Challenges with engaging stakeholders due to lack of anchoring	***Trialability*** – was integral to the PDSA structure and facilitated problem solving		
	***Compatibility***—the QIC structure facilitated compatibility by adapting improvement activities to different clinic settings	***Champions*** – presence of champions among the midwives facilitated the QIC	***Complexity*** – the QIC was complex due to high level of engagement required, and unclear purpose and roles in the initial phases		
	***Structural Characteristics*** – such as staff turnover, different routines and lack of time meant that some MHCs had a bigger need for specific routines to be established within the QIC	***Reflect and evaluate*** – opportunities to reflect and evaluate was provided by the PDSA and LS and was a facilitating factor			
	***Available Resources*** – Lack of time, mainly in the initial phases				

The constructs *structural characteristics* and *available resources* in the domain Inner setting*,* as well as *Planning* and *Engaging* within the domain Process are presented together in the result section due to interlinked findings

*Abbreviations*: *CFIR* Consolidated Framework for Implementation Research, *LS* Learning Seminar, *MHC* Maternal Health Clinic, *PDSA* Plan-Do-Study-Act, *QIC* Quality Improvement Collaborative

^a^Two constructs (*internal implementation leader* and *external change agent*) within the domain Process were merged into one construct (*project group*) because data extracts were found to have similar and interlinked meanings

^b^The construct *intervention source* within the domain Intervention characteristics has been renamed (*co-creation*) in order to better reflect meanings identified in the data

Below are the results presented for the five CFIR-domains and below each the adapted CFIR constructs in italics.

### Inner setting

#### *Compatibility* with the inner setting was facilitated by adaptability of the QIC

Facilitators perceived the QIC to be overall compatible with routine care because improvement activities could be adapted to the workflows in the different maternal health clinics. Adaptability, part of the domain intervention characteristics (presented below) was thus central to the domain inner setting as well. For example, application of the new routine to register data on contraceptive methods in the SPR was facilitated by evaluating the routine at the learning seminars and adapting it according to midwives’ input.

#### A favourable* learning climate* was created in the QIC

For example, midwives’ professional experiences were frequently emphasised as a crucial contribution to the QIC. Facilitators emphasised mutual knowledge sharing and that complementing views would create holistic solutions. They described their initiative to create an open, down-to earth and reflective atmosphere, for example by building trust and showing appreciation for midwives’ participation through informal conversations and providing meals. Data further indicated that when groups of midwives from different maternal health clinics came together and worked towards common goals, there was a tendency to want to highlight progress and an incentive to continue with their efforts.

It has brought up thoughts for improvement, what we can do better more clearly. Because you think that “these routine ways of working are all we have”, but you can actually always improve things. [Midwife, LS4]

#### *Communication* during and in between learning seminars varied

Communication during the learning seminars worked well and was closely connected to the beneficial learning climate. However, communication during action periods between facilitators and midwives was limited and described as a challenge due to lack of time for the midwives and not having a platform for regular communication.

#### Exchange of *information and knowledge* in three directions

The knowledge exchange was a crucial facilitating factor during the learning seminars, expressed both by midwives and facilitators. There was a knowledge exchange in three directions: i) between midwives regarding strategies, tips and reflections on how to apply improvement activities; ii) from facilitators to midwives regarding updated evidence on best practice contraceptive counselling postpartum and evidence on user feedback; and iii) from midwives to facilitators regarding context, implementation difficulties, women’s needs and resources.

#### Different *structural characteristics *and* available resources* at the maternal health clinics meant different needs

Staff turnover, lack of well-functioning routines to register information in SPR and lack of available resources and time posed barriers to apply the improvement activities. This was explained as a consequence of a generally stressful environment and that foreign-born women more often than native-born women required longer and somewhat more complex counselling due to interpreters and more detailed information due to reduced health literacy. Maternal health clinics facing these challenges to a larger extent had a bigger need for specific routines to be established within the QIC, for example regarding how and when to contact the women, compared to contexts that did not express these challenges.

### Process

#### An engaged and experienced* project group* ran the QIC

The facilitators were interested in the intervention and motivated to learn from both the midwives and other facilitators. The majority of them had extensive experience within their respective fields, including quality improvement projects and clinical experience. The researchers were described as an important support since they provided their experiences in QICs, evidence-based practices, and contributed a scientific perspective.

#### *Planning *and *engaging* with stakeholders at all levels

The importance of planning and engaging in order to succeed with QICs was emphasised. Two aspects of the planning phase caused challenges. Firstly, a new facilitator stepped into the leadership role just prior to the start of the QIC. Secondly, the project appeared not to be sufficiently anchored, and there were uncertainties regarding the goals and design of the project, leading to challenges with engaging stakeholders within the maternal health clinics. These uncertainties were identified and later resolved through dialogue at the learning seminars.

I felt that something got clearer today, we had a discussion and I think I understand why we were confused […] and with this knowledge I now understand that the goal was one thing, and the study was another. [Head of a maternal health clinic, LS2]

#### Presence of* champions* facilitated the QIC

The positive and reflective atmosphere during the learning seminars was facilitated by the participation of champions. Both primary and secondary data show that some midwives who participated in the QIC were especially engaged in the process and contributed to a greater extent with their reflections on challenges and possible improvements.

#### Opportunities to* reflect and evaluate* was provided by PDSA and the learning seminars

At the learning seminars, the project team provided continuous quantitative feedback through SPR data on progress towards goals for the respective maternal health clinics, as well as opportunity to discuss the user feedback and midwives’ experiences of applying the improvement activities. This led to critical reflections, ideas for further improvements, and solutions to the encountered problems.

### Intervention characteristics

#### *Co-creating* the QIC

The QIC was characterised by co-creation. It was partly internally developed, in terms of the active participation of midwives in planning and goal setting as well as the inclusion of the user-perspective when deciding upon the improvement activities. It was also externally developed, in terms of the pre-identified evidence-based areas for improvement presented by the project group.

I felt like we created health care, together. […] We provided suggestions “this is what we know is evidence-based, is it feasible?” […] And then they seized on most parts and extended it and designed it on the basis of their contexts*.* [Facilitator]

#### *Adaptability* of the project facilitated application of the improvement activities and inclusion of participant’s input

Adaptability was central to the QIC since the goal was to adapt the intervention regularly as part of the PDSA cycle. This meant that adaptability of the project facilitated compatibility with the inner setting, as described above. Midwives’ input on the application of the QIC was frequently and explicitly requested by facilitators throughout the QIC, individually and in groups, during the learning seminars.

#### *Trialability* facilitated problem solving

The PDSA structure made it possible to trial intervention components and reverse course if needed. Challenges encountered during action periods were resolved or discussed at the following learning seminars. For example, a point of discussion was how to register choice of contraception in a way that was both correct and feasible.

…we should document everything we do, why not this? We just have to learn. You have to get into the routine. – Yes, and that’s precisely what is so good I think, about this structure. It won’t be perfect right away, it takes time and we hope that we get there*.* [Discussion between midwife and facilitator, LS2]

#### A* complex* QIC, a high level of engagement required

The QIC was viewed as complex by the participants and facilitators, because the purpose of the intervention and the role of the participating midwives at the maternal health clinics was unclear. This was apparent mainly in the initial phases of the QIC and was closely related to the construct *planning*, which found that the QIC lacked a steady foundation in which all stakeholders were engaged in the project and understood the rationale and process. In general, the QIC required a high level of engagement from both participants and facilitators.

It perhaps requires more from the participants. It requires you to be active and make it the project your own. So if you expect to have everything served and not have to reflect by yourself, then it will be difficult. [Facilitator]

### Outer setting

#### *Patient needs and resource*s—especially important for foreign-born women

Some components of the QIC met specific challenges working with foreign-born women, due to needs that the current services could not meet. For example, time constraints, and difficulties calling women on the phone who do not speak Swedish. Furthermore, midwives expressed a feeling of working against common misconceptions or beliefs among women. Facilitators however perceived that critical awareness among midwives of women’s reasons behind declining a contraceptive method might be low.

It’s not so black and white. You have to always initiate a conversation about it. And that requires that you as a health care personnel have the ability to problematize […] that you can see the needs of the individual. [Facilitator]

### Individual characteristics

#### *Knowledge and beliefs—*lack of experience of QICs, but professional skills facilitated application of improvement activities

The overall knowledge of the process and practice of a QIC was low among midwives prior to the QIC. The familiarity on registration of data was however higher because register questions had been piloted. Midwives’ professional skills constituted an important source of knowledge because of their ability to adapt application of improvement activities to individual women.

## Discussion

This study presents novel findings regarding *how* and *why* a QIC was effective in improving contraceptive services in postpartum care together with foreign-born women. Most of the factors identified to have influenced the QIC’s ability to reach its intended goals were located in the CFIR domains: inner setting, intervention characteristics, and process.

Key facilitating factors contributing to the effectiveness of the QIC were adaptability and compatibility of improvement activities with routine care. Furthermore, there was a favourable learning climate that encouraged diverse perspectives and critical reflections among QIC participants, and the PDSA structure enabled trialability of improvement activities. This is in line with previous evidence suggesting that multidisciplinary teamwork in which participants are empowered to identify and implement possible improvements, are important to improve health outcomes in QICs [8–10]. This study also supports Wells et al.’s suggestion that the value of a QIC and its components may lie primarily in enabling a culture change in which all stakeholders collaborate to create change [1]. Furthermore, facilitating a learning climate and engaging all involved individuals in this QIC, could be understood as a way of negotiating the normative complexity inherent to quality improvement within healthcare [32]. By including learning seminars, with elements of everyday conversations or “catch-ups” between providers from different clinics, QICs has the potential to forge links between a diversity of perspectives on what should constitute ‘improvement’ and what is valuable in health care [32].

In terms of challenges, this study shows that crucial requirements for the success of a QIC are to acknowledge it as a complex process which requires a motivated and skilled project group, deliberate planning including efforts to engage stakeholders and anchor at all levels, and to include the perspective of users with lived experiences of contraceptive services. Findings from this study thus reiterated that a motivated and skilled project group with clear roles and expectations can facilitate a QIC, as well as support from experts in the field [8]. Ensuring that stakeholders are committed to the project and understand the process, and that there is sufficient time and resources available are factors that have been emphasised also in previous studies [1, 5–7].

In this study, contextual challenges such as lack of time were met by a favourable learning climate, adaptability and trialability of improvement activities, a motivated and skilled project group, and midwives’ professional skills and engagement. However, the distinction between which contextual factors belong to the “fixed” clinical context and which belong to the context of the QIC is not always clear. For example, midwives’ engagement can be part of their inherent motivations and interests, but also influenced by the project. As stated by Damschroder et al. [26], context consists of active variables that interacts with implementation. This study reiterates Dixon-Woods et al. [12] point that the collaborative nature does not automatically follow a QIC but needs to be supported by leadership and management techniques [1, 12].

### Methodological considerations

The major strength of this study is the combination of components from implementation science and improvement science. This study combines an analytical tool from the field of implementation science with data on health care practitioners’ experiences of applying a quality improvement tool in clinical practice [33]. This brought a more comprehensive understanding of *how* and *why a QIC* in the context of contraceptive services was effective and which factors influenced the QIC. Furthermore, this study increases the knowledge of how a framework commonly used in implementation science can be used to understand successful QICs in contraceptive services [33].

The main limitation of this study is lack of in-depth primary interview data with midwives, managers and other maternal health clinic staff. This type of data would have brought further depth to the findings, especially within the domain *individuals*. In addition, the potential subjectivity of facilitators risk bringing a set of preconceptions that could influence the accounts given by respondents.

Trustworthiness of the findings in this study is however enhanced through data and methodological triangulation including both participants and facilitators perspectives, data from learning seminars which were similar to focus groups, as well as individual in-depth interviews [34]. Confirmability was enhanced through a member check during which all respondents answered on the viability of the findings. Participant check with participating midwives could however not be applied due to time constraints. Dependability was increased by a transparent analysis process using a deductive approach with a well-developed theoretical framework, and by the use of methodological triangulation [34].

## Conclusions

The QIC’s effect on contraceptive services, targeting mainly foreign-born women, was due to the QIC being adaptable; encouraging all participants’ diverse perspectives, and with the possibility to trial and evaluate improvement activities. In order for future QICs to be successful it is crucial to view QIC as a complex process requiring high level of engagement and deliberate planning; to ensure the formation of a motivated and skilled project group; ensure adaptability and trialability of the QIC and the improvement activities; and to facilitate a favourable learning climate that emphasizes co-creation and mutual learning. The majority of the factors that influenced the QIC’s possibility reach its intended goals were found inside the QIC setting and concerned intervention characteristics and process, or the inner setting of the QIC project group and the maternal health clinics.

### Supplementary Information

**Additional file 1. **Consolidated Framework for Implementation Research Constructs.

**Additional file 2. **Interview guide - QIC, primary data collection.

## Data Availability

The dataset of secondary data generated and/or analysed during the current study is not publicly available due to restrictions from the ethics review board but can be made available to qualified researchers upon request, after approval from the ethics board. EL should be contacted to request the secondary data. ISH should be contacted to request the primary data.

## Abbreviations

QIC: Quality Improvement Collaborative
CFIR: Consolidated Framework for Implementation Research
SRHR: Sexual and Reproductive Health and Rights
SPR: Swedish Pregnancy Register
PDSA: Plan-Do-Study-Act
