# Supplementary material for: What factors influence a Quality Improvement Collaborative in improving contraceptive services for foreign-born women? A qualitative study in Sweden
Source: BMC Health Serv Res. 2023 Oct 11;23:1089. doi: 10.1186/s12913-023-10060-2 (PMC10568973; doi:10.1186/s12913-023-10060-2)
Supplement: Supplementary file 2 — Additional file 2. Interview guide - QIC, primary data collection. [file 12913_2023_10060_MOESM2_ESM.docx]

**Interview guide - QIC, primary data collection**

*Translated from Swedish.*

Main probe: How do you think that affected the project?

- Could you start by describing your role in the project?
- How would you describe your experience of working with the QIC method in general?

**The QIC method and its process (CFIR domains intervention characteristics and process)**

- What adjustments had to be done in order for the QIC method and the registration of performance data in SPR to fit into and function in the context, if any?
- How complicated would you say that the QIC method is from your experience of participating in the project? (Prompt: scope, complexity, difference between the structure of the method and routine work)
- Which similar or related project, program or processes were ongoing in the time surrounding the project, if any?
- Do you think that the method was implemented according to plan? (Probe: If yes, how? If no, why?)

**Outer and inner context (CFIR domains outer and inner setting)**

- Did you experience that any policies, guidelines or management objectives was influencing the project? (Prompt: financial or other incitements)
- Did you experience that any organizational factors within the clinics were influencing the project? (Prompt: Size, physical environment, information systems, IT-systems, workplace culture).
  1. How was the atmosphere during the learning seminars?
  2. How did you experience the workplace culture among the participating midwives at the clinics?
- If you consider contraceptive counselling, how well would you say the QIC method fit into the workflows and practices of that context, based on your experience?
  1. Have you worked with any other quality improvement method that worked as well or better in this context?
- Were there any internal or external change you think could have facilitated the QIC-project?

**Individuals (CFIR domain individuals)**

- Was there anyone within or outside the project that was particularly important for the implementation?
- What role did the heads of the maternal health clinics play in the implementation? What support did you receive from them?
- How did you experience the leadership from the researchers?
- What motivated you in the work with the project?
